# Changes in gastrocnemius MTU stiffness and their correlation with plantar pressure in patients with knee osteoarthritis

**DOI:** 10.3389/fbioe.2024.1378031

**Published:** 2024-05-09

**Authors:** Wenjing Wang, Feng Li, Jiayi Guo, Zhijie Zhang

**Affiliations:** ^1^ Department of Sport Rehabilitation, Shanghai University of Sport, Shanghai, China; ^2^ Rehabilitation Therapy Center, Luoyang Orthopedic Hospital of Henan Province, Orthopedic Hospital of Henan Province, Luoyang, China

**Keywords:** Achilles tendon, gastrocnemius, knee osteoarthritis, plantar pressure, shear wave elastography, stiffness

## Abstract

**Background:**

Abnormal, excessive, and repetitive knee load is a critical risk factor for osteoarthritis (OA). The gastrocnemius muscle-tendon unit (MTU) interacts with foot biomechanics and is vital in cushioning the knee load. Abnormal gastrocnemius activation and plantar pressure during walking in patients with knee OA may negatively affect gastrocnemius MTU stiffness, increasing knee load. Few studies investigated the relationship between gastrocnemius MTU stiffness and plantar pressure. This study aimed to evaluate the changes in gastrocnemius MTU stiffness in patients with knee OA and their correlations with plantar pressure and clinical symptoms.

**Methods:**

Thirty women patients with unilateral knee OA and 30 healthy women participants were recruited. Shear wave elastography was used to quantify gastrocnemius MTU stiffness in ankle resting and anatomical 0° positions, defined as natural and neutral positions in this study. A plantar pressure analysis system was used to collect the plantar pressure parameters on the symptomatic side in patients with knee OA. The Western Ontario and McMaster Universities Osteoarthritis Index (WOMAC) and Visual Analogue Scale (VAS) scores were used to measure the severity of clinical symptoms.

**Results:**

Medial and lateral gastrocnemius (MG and LG) stiffness on both the asymptomatic and symptomatic sides in patients with knee OA was increased compared with that in healthy participants. The MG and LG optimal cutoff stiffness in the natural position was 15.73 kPa and 14.25 kPa, respectively. The optimal cutoff stiffness in the neutral position was 36.32 kPa and 25.43 kPa, respectively, with excellent sensitivity and specificity. The MG and LG stiffness were positively correlated with the percentages of anterior and medial plantar pressure and negatively correlated with the length of pressure center path. The LG and MG were significantly correlated with WOMAC and VAS scores.

**Conclusion:**

Patients with knee OA have increased gastrocnemius muscle stiffness, closely related to plantar pressure and clinical symptoms. Monitoring the gastrocnemius muscle in patients with knee OA can provide an essential basis for its prevention and treatment.

## 1 Introduction

Knee osteoarthritis (OA) is a chronic disease characterized by cartilage degeneration and hyperostosis (Lespasio et al., 2017). More than 60% of patients with knee OA self-report knee instability, and quadricep weakness may be a significant risk factor (Neelapala, 2019). The quadriceps and gastrocnemius muscle (GM) are crucial for maintaining knee stability and cushioning joint load (Shelburne et al., 2006). The GM works cooperatively with the quadriceps to stabilize the knee by keeping the tibia in an anterior position (Kvist and Gillquist, 2001). Co-activation of GM and other muscles around the knee can improve knee instability by increasing tibiofemoral joint compression, stiffness, and proprioception (Wilk et al., 1999). Previous studies found abnormal activation patterns of GM during weight bearing in patients with knee OA (Cabral et al., 2021). Specifically, the GM stabilizes the knee through co-activation with the tibialis anterior muscle during squatting and reduces the medial knee laxity through co-activation with the medial quadriceps during walking (Childs et al., 2004; Lewek et al., 2004). As knee flexors, the abnormal activation of GM can affect the knee adduction moment and change the mechanical properties of soft tissue, which is closely associated with disease development.

The gastrocnemius muscle-tendon unit (MTU) may also indirectly affect lower-limb biomechanics through interactions with foot posture. The feet absorb ground reaction forces and shape the postural alignment and joint movement patterns (Butler et al., 2007). Changes in foot posture can cause an abnormal load on the lower limbs and may be a risk factor for foot injury and knee OA (Paterson et al., 2017). Patients with knee OA have abnormal foot postures during the gait cycle, such as flatfoot and excessive pronation, which are closely associated with the clinical symptoms (Gross et al., 2011). Excessive foot pronation may compensate for limited ankle dorsiflexion and affect the anterior and medial plantar pressures (Zhang et al., 2017). Previous studies have found that GM contractions increase the percentage of anterior and middle plantar pressure and decrease posterior pressure (Aronow et al., 2006). Individuals with excessive pronation and medial plantar pressure have a higher risk of GM pain after running (Willems et al., 2007).

Previous studies showed that increased quadriceps and hamstring stiffness are associated with clinical symptoms in patients with knee OA (Chang et al., 2021; Li et al., 2021). A recent study reported the relationship between gastrocnemius MTU stiffness and foot posture asymmetry in patients with OA using a non-invasive handheld machine (MyotonPRO) (Chen et al., 2021). However, it did not compare the gastrocnemius MTU stiffness with healthy adults. The changes in gastrocnemius MTU stiffness during knee OA progression are unclear. Moreover, to our knowledge, no study has investigated the relationship between gastrocnemius MTU stiffness and plantar pressure. As a non-invasive device, shear wave elastography (SWE) can quantitatively evaluate deeper soft tissue stiffness compared to MyotonPRO regardless of adipose tissue thickness and shows good inter- and intra-observer reliability for stiffness evaluation (Zhou et al., 2019). In recent years, SWE has gradually emerged in studying musculoskeletal diseases, which can identify specific hamstring and iliotibial band lesions and provide technical support for auxiliary diagnosis of knee OA (Li et al., 2021; Yagi et al., 2023). Quantitative evaluation of gastrocnemius MTU stiffness and its relationship with foot biomechanics in patients with knee OA using SWE is necessary for more comprehensive mechanical properties of soft tissue around the knee, which is beneficial for accurate diagnosis and individualized treatment in patients with knee OA.

Thus, the purpose of this study was to 1) explore the change in gastrocnemius MTU stiffness in patients with knee OA, 2) examine the correlation between gastrocnemius MTU stiffness and dynamic plantar pressure, and 3) evaluate the correlation between gastrocnemius MTU stiffness and clinical symptoms in patients with knee OA. We hypothesized that the patients with unilateral knee OA have significantly increased gastrocnemius MTU stiffness on both symptomatic and asymptomatic sides compared with healthy adults, and the gastrocnemius MTU stiffness on the symptomatic side is associated with dynamic plantar pressure and clinical symptoms.

## 2 Methods

### 2.1 Participants

Sixty women (30 healthy participants and 30 patients with unilateral knee OA) volunteered to participate in the study (Table 1). We invited patients preliminarily diagnosed with unilateral knee OA at the Osteoarthropathy Department of Luoyang Orthopedic Hospital in Henan Province to participate in this study from July to October 2023. The inclusion criteria for patients with knee OA were as follows: 1) women aged ≥50 years; 2) diagnosed with unilateral knee OA according to American College of Rheumatology classification (Sharma, 2021); 3) Kellgren and Lawrence grade 2 or 3 (Kellgren and Lawrence, 1957); and 4) no skin lesions in the measurement regions. The exclusion criteria were as follows: 1) acute or infectious diseases; 2) other neurological or musculoskeletal diseases affecting the lower tissues, such as stroke, fracture, and rheumatoid arthritis; 3) severe cognitive impairment or mental illness; 4) use of drugs that affect the lower tissues or anti-inflammatory analgesics; and 5) recent rehabilitation treatment. The demographic characteristics of healthy individuals recruited for this study were matched to those of patients with knee OA. Healthy individuals with a history of musculoskeletal or neurological disorders were excluded. All participants were required to avoid physical activity for 48 h before the experiment. This study was approved by the Ethics Committee of Luoyang Orthopedic Hospital of Henan Province (approval date: 17 March 2023, approval number: KY 2023-008-010). This study adhered to the principles of the Declaration of Helsinki. All participants voluntarily signed an informed consent form prior to the experiment.

**TABLE 1:** Characteristics of participants.

	Patients with knee OA (*n* = 30)	Healthy participants (*n* = 30)	*p*
Age (years)	59.73 ± 4.83	61.33 ± 7.17	0.625
Height (m)	1.59 ± 0.05	1.60 ± 0.04	0.778
Body mass (kg)	63.14 ± 7.25	60.60 ± 7.56	0.189
BMI (kg/m^2^)	24.83 ± 2.66	23.70 ± 2.49	0.094
Pain (WOMAC)	6.53 ± 1.85	—	—
Stiffness (WOMAC)	2.77 ± 0.94	—	—
Physical function (WOMAC)	18.33 ± 5.50	—	—
WOMAC	27.67 ± 7.63	—	—
VAS	5.4 ± 1.38	—	—

Data are presented as mean ± standard deviation.

BMI, body mass index; OA, osteoarthritis; VAS, visual analogue scale; WOMAC, Western Ontario and McMaster Universities Osteoarthritis Index.

### 2.2 Procedures

All tests were performed in a quiet treatment room with an ambient temperature of 25°C. Demographic characteristics such as sex, age, height, and body mass were collected before the experiment. Patients with knee OA also completed the Western Ontario and McMaster Universities Osteoarthritis Index (WOMAC) and Visual Analogue Scale (VAS). The WOMAC scale contains 24 questions with a score of 0–96 and is divided into 3 subscales: pain, stiffness, and physical function. The pain subscale score ranges from 0 to 20; stiffness, 0 to 8; and physical function, 0 to 68. Higher scores indicate more severe clinical symptoms (Bellamy et al., 1988). The VAS is scored on a 10-point scale, with higher scores indicating more severe pain (Hawker et al., 2011). The WOMAC and VAS are effective and reliable for evaluating patients with knee OA.

Medial gastrocnemius (MG), lateral gastrocnemius (LG), and Achilles tendon (AT) stiffness were measured sequentially in the natural and neutral positions by SWE. Firstly, the participants lay prone on the treatment bed with their hips and knees fully extended and their feet hanging freely from the bed. The gastrocnemius MTU stiffness in the natural position was measured under this condition. Then, the ankle was fixed at 90° with a brace for the neutral position. The participants were asked to avoid active muscle contractions during the measurements. The operator also monitored muscle contractions indirectly through ultrasound images. If the image shows a significant fluctuation, incomplete muscle relaxation was assumed. For patients with knee OA, stiffness was assessed on both symptomatic and asymptomatic sides. A previous study reported no difference in gastrocnemius MTU stiffness between dominant and non-dominant sides in healthy adults (Chang et al., 2020). Thus, healthy participants were evaluated only on the dominant side, which was defined as the side they used when kicking a ball (Lenskjold et al., 2015). The measurements were repeated 3 times for each tissue, and the average was calculated. Prior to the experiment, the measurement regions were marked to ensure that each measurement was in the same position and to avoid errors.

A plantar pressure analysis system was used to collect plantar pressure parameters on the symptomatic side in patients with knee OA during walking. The core component of the device is a 515 mm long by 380 mm wide force plate with a frequency of 300 Hz and contains 6,144 sensors. The force plate was placed at the center of a 300 cm long by 60 cm wide walkway and connected to a computer with plantar pressure acquisition and analysis software. Before the formal assessment, the participants were asked to practice walking comfortably barefoot for 5 min along the walkway. Then, based on practice, a suitable starting position was determined to ensure they could step in the center of the force plate at the third step in normal walking. The participants were asked to walk normally three times along the walkway without focusing their eyes on the plate in the formal test. There was no rest between each time. The experimenter instructed the patients to perform a plantar pressure test and recorded the parameters.

### 2.3 Shear wave elastography

**FIGURE 1:**
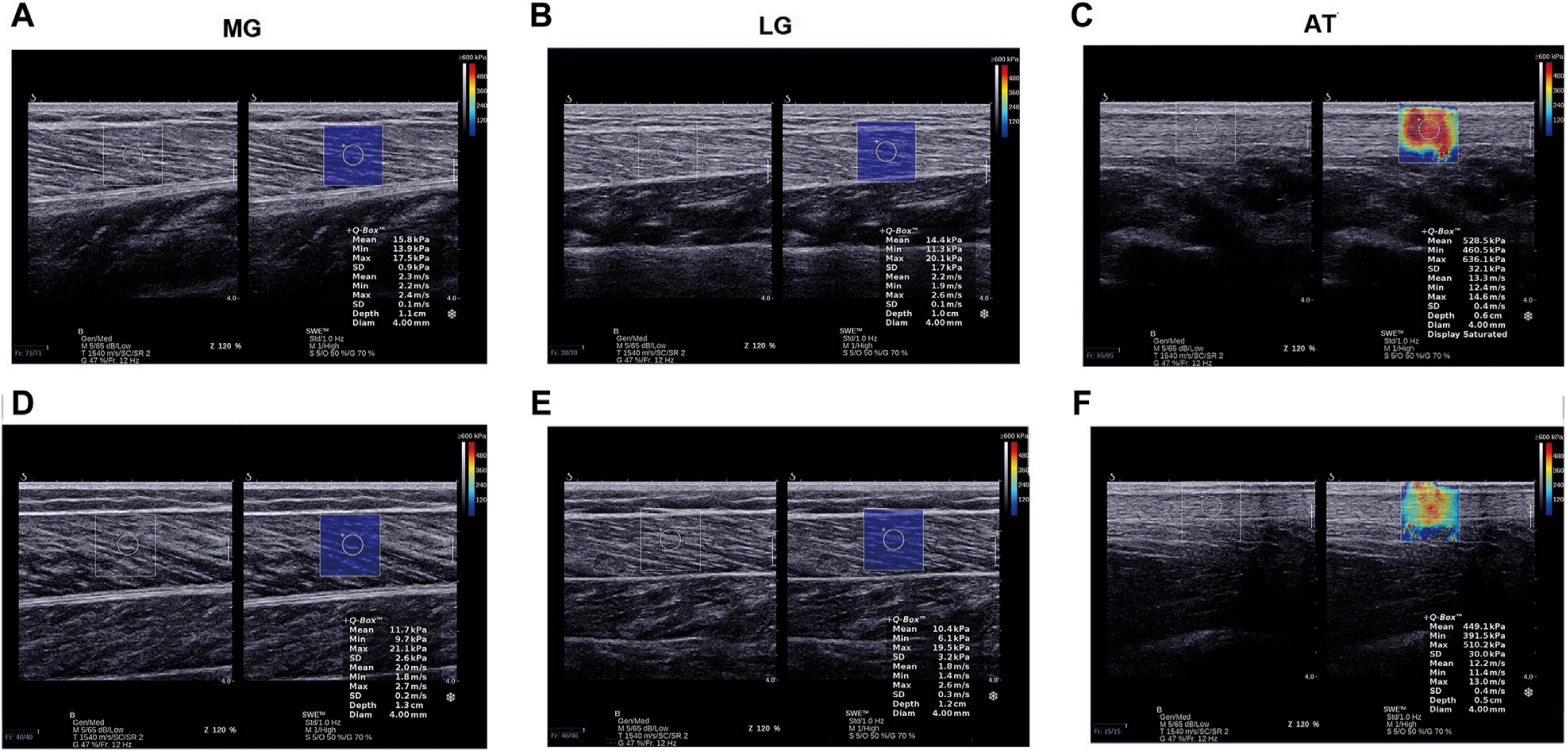
Examples of shear modulus maps of MG, LG, and AT in patients with knee OA **(A–C)** and healthy participants **(D–F)**. AT, Achilles tendon; LG, lateral gastrocnemius; MG, medial gastrocnemius; OA, osteoarthritis.

An ultrasonographic device (Aixplorer, Supersonic Imagine, Aix-en-Provence, France) with a 4–15 MHz linear transducer array (SL15-4, Supersonic Imagine, Aix-en-Provence, France) was used to quantitatively evaluate gastrocnemius MTU stiffness (Dietrich and Dong, 2016). The device modes were selected for musculoskeletal mode and SWE mode. The device parameters were set as follows: conventional preset enhancement mode, 4.0 cm scanning depth, 50% opacity of images, and 0–600 kPa measurement range. The stiffness shown in the image is the average within the region of interest (ROI). The ROI of MG and LG was a 4 mm diameter circle. The ROI diameter of the AT was determined based on the tissue thickness (Liu et al., 2020). The probe was placed vertically on the skin and longitudinally parallel to the muscle fibers without applying pressure for measurement. The probe contacted the skin using a water-soluble coupler to avoid interference with the image. MG and LG stiffness were measured at nearly 30% of the distance from the popliteal fossa to the malleolus (Zhou et al., 2019). The AT stiffness was measured 4 cm above the calcaneal tubercle. When an image was recognized, the probe was held in the measurement region for >5 s to obtain a stable image. The colored shear modulus maps of MG, LG, and AT are shown in Figure 1, where red represents a high shear modulus and blue represents a low value (Ferraioli et al., 2012). The shear modulus is calculated based on the shear wave velocity (m/s) generated within the tissue, and the formula is as follows:

$$\mu =\rho c^{2}$$

μ is the shear modulus, ρ is the material density, and c is the wave speed (Sigrist et al., 2017). In this study, tissue stiffness was replaced by the shear modulus. A strong linear correlation between the shear modulus and stiffness has been demonstrated when the probe is parallel to the tissue fibers (Eby et al., 2013). A higher shear modulus indicates stiffer tissue.

### 2.4 Plantar pressure

Plantar pressure parameters were automatically measured by the plantar pressure analysis system, including the percentages of center of pressure (COP) path length (Long%), anterior plantar pressure (APP%), and medial plantar pressure (MPP%), as well as the center of pressure excursion index (CPEI) and COP speed. The Long% is the percentage of COP path length to foot length (Saito et al., 2013). The foot is divided into three parts: anterior, middle, and posterior. APP% is the percentage of the anterior plantar pressure to the total anterior and posterior pressure. The foot midline from the heel centroid to the forefoot between the second and third metatarsal heads divides the plantar pressure into medial and lateral parts. MPP% is the medial plantar pressure percentage to the total pressure (Li et al., 2023). The CPEI is calculated by drawing a line connecting the first and last points in the COP path and then measuring the distance between this line and the COP in the distal third of the foot length as a percentage of the foot width. A lower CPEI indicates a more pronated foot, whereas a higher CPEI indicates a more supinated foot during walking (Galica et al., 2013).

### 2.5 Statistical analysis

SPSS 26.0 software (version 26.0, IBM, United States) was used for statistical analysis. All statistical data were presented as mean ± standard deviation. The Shapiro–Wilk test was used to analyze normality. Levene’s test was used to describe the homogeneity of variance in the data. Demographic characteristics between groups were compared using an independent sample t-test. The difference in gastrocnemius MTU stiffness was assessed using one-way ANOVA. Bonferroni tests were used to conduct multiple comparisons after the event if statistical significance was observed. A non-parametric test was used to analyze data when continuous variables were non-normal or showed heterogeneity of variance. Receiver operating characteristic (ROC) curves were used to determine the cutoff point for gastrocnemius MTU stiffness. The Youden’s index (J) was calculated based on sensitivity and specificity.

J=sensitivity+specificity−1

Stiffness was the best cutoff point for identifying knee OA when Youden’s index was highest (Leong et al., 2016). The Pearson correlation coefficient (r) was used to describe the correlation between the gastrocnemius MTU stiffness, plantar pressure parameters, and scale scores. Spearman’s correlation coefficient was used to analyze the correlation between non-normal data. The r value of 0–0.2 indicates very weak or no correlation, 0.2–0.4 is weak, 0.4–0.6 is moderate, 0.6–0.8 is strong, and 0.8–1.0 is solid. *p* < 0.05 was considered statistically significant.

## 3 Results

### 3.1 Participant characteristics

We recruited 30 patients with knee OA (59.73 ± 4.83 years) and 30 healthy participants (61.33 ± 7.17 years) in the study (Table 1). There was no significant difference in the body mass index (BMI) between groups (24.83 ± 2.66 kg/m^2^ vs. 23.70 ± 2.49 kg/m^2^). For patients with knee OA, the WOMAC score was 27.67 ± 7.63 (pain: 6.53 ± 1.85; stiffness: 2.77 ± 0.94; physical function: 18.33 ± 5.50), and the VAS score was 5.4 ± 1.38.

### 3.2 Gastrocnemius MTU stiffness between the patients with knee OA and healthy participants

**FIGURE 2:**
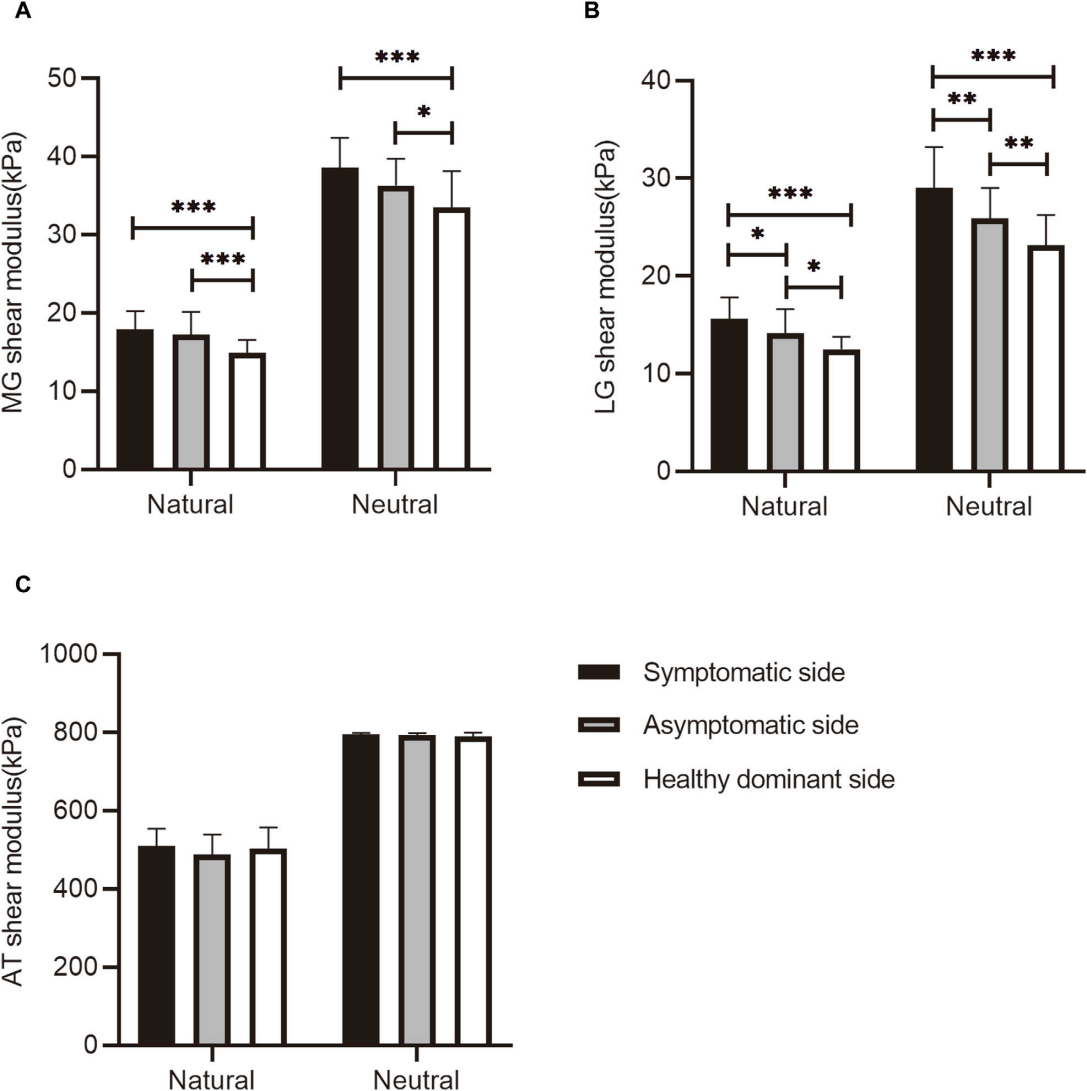
The **(A)** MG, **(B)** LG, and **(C)** AT shear modulus at different positions in healthy participants and patients with knee OA. *p < 0.05, **p < 0.01, ***p < 0.001, significant difference compared with healthy participants. AT, Achilles tendon; LG, lateral gastrocnemius; MG, medial gastrocnemius; OA, osteoarthritis.

The gastrocnemius MTU stiffness in patients with knee OA and healthy participants is shown in Figure 2. MG and LG stiffness in patients with knee OA on both the symptomatic and asymptomatic sides increased in the natural and neutral positions compared with that in healthy participants (*p* < 0.05). The LG stiffness on the symptomatic side was significantly higher than that on the asymptomatic side (*p* < 0.05). However, the MG stiffness on the symptomatic and asymptomatic sides was symmetrical (*p* > 0.05). There was no significant difference in the AT stiffness between healthy participants and patients with knee OA (*p* > 0.05).

### 3.3 Optimal cutoff points for identifying patients with knee OA

**FIGURE 3:**
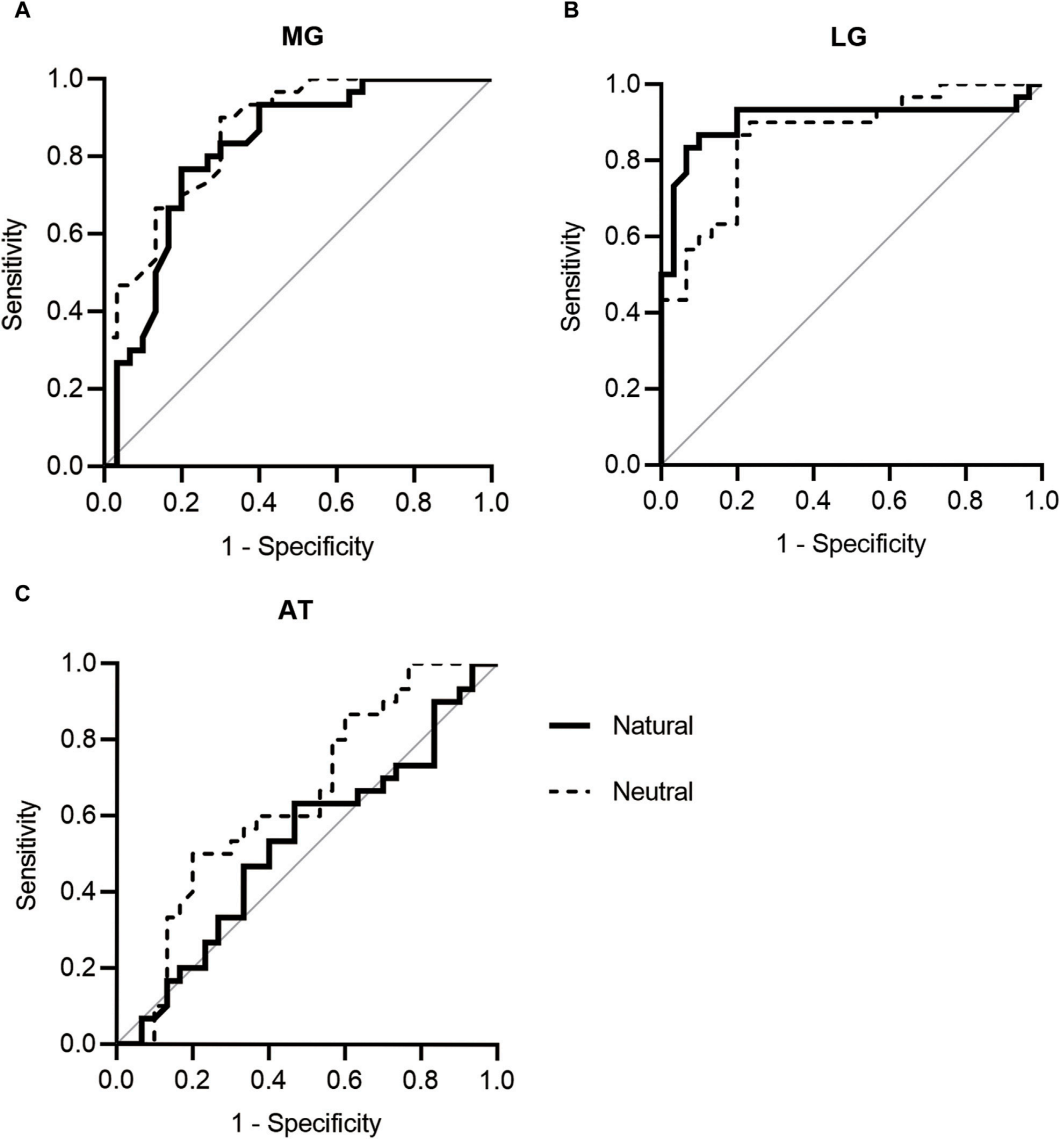
ROC curves of **(A)** MG, **(B)** LG, and **(C)** AT stiffness. AT, Achilles tendon; LG, lateral gastrocnemius; MG, medial gastrocnemius; ROC, receiver operating characteristic.

The optimal cutoff points for MG and LG stiffness at the natural and neutral positions for identifying patients with knee OA were determined by constructing ROC curves (Figure 3). The area under the ROC curve (AUC) of the MG at the natural and neutral positions was 0.863 and 0.817, respectively. Youden’s index showed that the sensitivity and specificity of 15.73 kPa MG stiffness at the natural position in identifying patients with knee OA were 0.900 and 0.700, respectively. The 36.32 kPa in the neutral position had a sensitivity of 0.767 and specificity of 0.800. For LG stiffness, the AUC of the LG at the natural and neutral positions was 0.906 and 0.864, respectively. The optimal cutoff point was 14.25 kPa at the natural position, and the sensitivity and specificity for identifying patients with knee OA were 0.833 and 0.933, respectively. The 25.43 kPa in the neutral position had a sensitivity of 0.867 and specificity of 0.800. The AUCs for AT in the natural and neutral positions were not statistically significant.

### 3.4 Correlations between gastrocnemius MTU stiffness and dynamic plantar pressure

The correlation coefficients between the gastrocnemius MTU and plantar pressure are listed in Table 2. In patients with knee OA, there was a significant negative correlation between LG stiffness and gait line ratio at the natural position (r = −0.398, *p* = 0.029). LG stiffness was positively correlated with anterior and medial plantar pressure in the natural position, with correlation coefficients of 0.450 and 0.602, respectively. At the neutral position, MG stiffness and LG stiffness were negatively correlated with the gait line ratio, and the correlation coefficients were −0.462 and −0.405, respectively. A significant negative correlation was observed between MG stiffness and COP speed (r = 0.435, *p* = 0.016). GM stiffness was positively correlated with anterior plantar pressure (MG: r = 0.382, *p* = 0.037; LG: r = 0.409, *p* = 0.025). There was also a significant positive correlation between GM stiffness and medial plantar pressure (MG: r = 0.422, *p* = 0.020; LG: r = 0.463, *p* = 0.010). However, no significant correlation was found between the AT stiffness and plantar pressure.

**TABLE 2:** Correlations between gastrocnemius MTU stiffness and dynamic plantar pressure in patients with knee OA.

	Natural			Neutral		
	MG	LG	AT	MG	LG	AT
Long%						
r	−0.097	−0.398*	−0.009	−0.462*	−0.405*	−0.183
p	0.612	0.029	0.962	0.010	0.027	0.333
APP%						
r	0.133	0.450*	0.076	0.382*	0.409*	−0.025
p	0.484	0.013	0.691	0.037	0.025	0.897
MPP%						
r	0.256	0.602**	0.191	0.422*	0.463*	−0.293
p	0.173	<0.001	0.312	0.020	0.010	0.116
CPEI						
r	−0.264	−0.319	−0.348	−0.320	−0.304	−0.222
p	0.159	0.086	0.060	0.085	0.102	0.239
COP speed						
r	−0.092	−0.120	0.028	−0.435*	−0.297	−0.041
p	0.630	0.528	0.883	0.016	0.111	0.831

The correlation coefficient (r) value is between −1 and 1, with closer to 1 indicating a stronger positive correlation and closer to −1 indicating a stronger negative correlation.

*p < 0.05, **p < 0.001, significant correlations between gastrocnemius MTU stiffness and plantar pressure on the symptomatic side.

APP%, percentage of anterior plantar pressure; AT, Achilles tendon; COP, center of pressure; CPEI, center of pressure excursion index; LG, lateral gastrocnemius; Long%, percentage of COP path length; MG, medial gastrocnemius; MPP%, percentage of medial plantar pressure; MTU, muscle-tendon unit; OA, osteoarthritis.

### 3.5 Correlations between gastrocnemius MTU stiffness and clinical symptoms

In the natural position, LG stiffness was positively correlated with the WOMAC and VAS scores (Table 3). MG stiffness and AT stiffness were positively correlated with the WOMAC total score and subscale scores for pain and physical function, and were also significantly correlated with the VAS score. They were not significantly correlated with the stiffness subscale scores. MG and LG stiffness were also positively correlated with WOMAC and VAS scores in the neutral position. No significant correlation was found between AT stiffness and scores.

**TABLE 3:** Correlations between gastrocnemius MTU stiffness and clinical symptoms in patients with knee OA.

	Natural			Neutral		
	MG	LG	AT	MG	LG	AT
Pain (WOMAC)						
r	0.557**	0.620**	0.465**	0.774**	0.633**	0.057
p	0.001	<0.001	0.010	<0.001	<0.001	0.767
Stiffness (WOMAC)						
r	0.275	0.430*	0.307	0.427*	0.511**	−0.087
p	0.141	0.018	0.099	0.019	0.004	0.648
Physical function (WOMAC)						
r	0.403*	0.759**	0.434*	0.572**	0.539**	0.171
p	0.027	<0.001	0.017	0.001	0.002	0.367
WOMAC						
r	0.461*	0.770**	0.468**	0.662**	0.591**	0.123
p	0.010	<0.001	0.009	<0.001	0.001	0.516
VAS						
r	0.541**	0.629**	0.396*	0.555**	0.648**	0.340
p	0.002	<0.001	0.031	0.001	<0.001	0.066

The correlation coefficient (r) value is between −1 and 1, with closer to 1 indicating a stronger positive correlation and closer to −1 indicating a stronger negative correlation.

*p < 0.05, **p < 0.001, significant correlations between gastrocnemius MTU stiffness and clinical symptoms on the symptomatic side.

AT, Achilles tendon; LG, lateral gastrocnemius; MG, medial gastrocnemius; MTU, muscle-tendon unit; OA, osteoarthritis; VAS, visual analogue scale; WOMAC, Western Ontario and McMaster Universities Osteoarthritis Index.

## 4 Discussion

This study investigated the changes in passive gastrocnemius MTU stiffness and their correlation with dynamic plantar pressure, WOMAC, and VAS scores in patients with knee OA. These results are consistent with our hypothesis that patients with unilateral knee OA have increased MG and LG stiffness on the symptomatic side, significantly associated with low Long%, high APP%, high MPP%, and severe clinical symptoms. Patients with unilateral knee OA also showed a significant increase in MG and LG stiffness on the asymptomatic side compared with healthy participants. More importantly, this study identified the optimal cutoff points for MG and LG stiffness for recognizing knee OA in the natural and neutral positions. Increased AT stiffness was also associated with severe clinical symptoms, although the stiffness was not significantly different between healthy participants and patients with knee OA.

This is the first study to explore the difference in gastrocnemius MTU stiffness between patients with knee OA and healthy adults. In the natural position, the MG and LG stiffness on the symptomatic side increased by 20% and 25%, respectively, and that on the asymptomatic side increased by 14% and 13%, respectively, compared with those in the control group. In the neutral position, the MG and LG stiffness on the symptomatic side increased by 15% and 25%, respectively, and that on the asymptomatic side increased by 8% and 12%, respectively. The increased MG and LG stiffness in patients with knee OA may be attributed to abnormal muscle activation during physical activity (Childs et al., 2004; Lewek et al., 2004). A previous study reported that the MG is activated in patients with severe knee OA during most of the gait cycle (Astephen et al., 2008). The MG and LG are essential in shaping lower limb movement patterns. The increased MG and LG stiffness in patients with knee OA can reduce the knee range of motion (ROM), increasing knee load and accelerating disease progression (Hirata et al., 2020). Clinicians and therapists should pay attention to changes in MG and LG stiffness in patients with knee OA and develop targeted programs to reduce stiffness. Moreover, the MG and LG stiffness assessed with SWE showed good sensitivity, specificity, and accuracy in identifying the symptomatic side in patients with unilateral knee OA, implying that SWE may be a potential diagnostic method for knee OA.

Additionally, MG and LG stiffness on the asymptomatic side was higher in patients with unilateral knee OA than in healthy adults. Patients with unilateral knee OA may place an increased load on the asymptomatic side while standing and walking to reduce pain and compensate for completing physical activities. This finding suggests that most patients with unilateral knee OA progress to bilateral within 12 years (Metcalfe et al., 2012). The physical function of patients with bilateral knee OA is significantly worse than that of patients with unilateral knee OA, which predicts a more severe medical and financial burden on families and society with bilateral knee OA. Clinicians can identify the risk of diseases and intervene early through the quantitative evaluation of soft tissue stiffness using SWE. More attention should be paid to the functional assessment of the asymptomatic side during the diagnosis and follow-up of unilateral knee OA to prevent bilateral occurrence.

This study first found that passive MG and LG stiffness negatively correlated with Long% and positively correlated with APP% and MPP%. The mechanical properties of the gastrocnemius MTU interact with the plantar pressure. Previous studies have also reported the correlation between the gastrocnemius MTU and plantar pressure. Aronow et al. (2006) showed that APP% and MPP% increased as MG and LG tension, whereas the percentage of posterior pressure decreased. A prospective study found that individuals with exercise-related MG and LG pain had increased pronation deviation and medial plantar pressure during shot running compared with healthy adults, suggesting that changes in foot posture caused by MG and LG stiffness may negatively affect the soft tissue (Willems et al., 2007). The association of MG and LG with plantar pressure may be established by limited ankle ROM. Previous studies showed that the MG and LG stiffness was negatively associated with passive ankle dorsiflexion ROM, which can lead to earlier heel-off and longer forefoot support time in the gait cycle, increasing APP% (Hirata et al., 2020). On the other hand, limited ankle dorsiflexion can lead to compensatory foot pronation, prolonging peak force time in medial plantar pressure, and then the MPP% may increase (Dodelin et al., 2020). Increased MG and LG stiffness and abnormal dynamic plantar pressure are related to the knee load (Schwachmeyer et al., 2015). These findings suggest that the abnormal distribution of plantar pressure can be improved clinically by reducing the MG and LG stiffness, decreasing the knee load, and delaying the progression of the disease. Adjusting plantar pressure distribution during walking may be feasible to reduce the MG and LG stiffness in patients with knee OA.

This study found that gastrocnemius MTU stiffness on the symptomatic side were significantly correlated with WOMAC and VAS scores. Previous studies also observed that soft tissue stiffness was correlated with the clinical symptoms of musculoskeletal diseases. The MG stiffness was positively correlated with pain in patients with plantar fasciitis (Zhou et al., 2020). AT stiffness gradually increased after reconstruction and was positively correlated with functional scores (Zhang et al., 2016). The WOMAC and VAS scores reflect the severity of the disease and are commonly used to evaluate clinical efficacy in patients with knee OA. Wang et al. (2016) explored the effects of combined whole-body vibration and quadriceps resistance exercises on physical function in patients with knee OA. These patients had lower VAS and WOMAC scores than those performing only quadriceps resistance exercise. The combination of whole-body vibration and quadriceps resistance exercises showed good outcomes. The prognosis of knee OA may be improved by exploring treatments to reduce gastrocnemius MTU stiffness and correct abnormal foot posture.

This study had some limitations. First, we did not monitor the muscles in real time using electromyography. Although the participants were asked to relax during the experiment, the influence of muscle contraction on stiffness could not be eliminated. The gastrocnemius MTU stiffness may be overestimated. This study only assessed the superficial tissues. Soleus stiffness was not evaluated because of the limited penetration of SWE. The stiffness threshold obtained using SWE in this study was 800 kPa. The AT stiffness in some participants in the neutral position exceeded this value, and it was found in both patients with knee OA and healthy adults. The AT stiffness exceeding the threshold is capped at 800 kPa. There may be errors in the results based on AT stiffness in the neutral position. Moreover, due to the lack of plantar pressure in healthy adults, the association between gastrocnemius MTU stiffness and plantar pressure in healthy adults is unclear, and this study could not determine the difference in association between patients with knee OA and healthy adults. Finally, gastrocnemius MTU stiffness is affected by physiological factors such as age and sex, and the cutoff point determined in this study can only be applied to women with unilateral knee OA over 50 years of age.

## 5 Conclusion

GM stiffness increased in patients with knee OA and was correlated with plantar pressure and clinical symptoms, which provides a theoretical basis for the precise clinical diagnosis and treatment. Clinicians can improve foot biomechanics and relieve clinical symptoms by reducing GM stiffness. Patients with unilateral knee OA have increased GM stiffness on the asymptomatic side and are at risk of developing bilateral knee OA in the future. Clinicians should pay attention to the GM stiffness on the asymptomatic side to prevent the occurrence of bilateral knee OA.

## Data Availability

The original contributions presented in the study are included in the article/supplementary material, further inquiries can be directed to the corresponding author.
